# Non-Hemagglutinating Flaviviruses: Molecular Mechanisms for the Emergence of New Strains via Adaptation to European Ticks

**DOI:** 10.1371/journal.pone.0007295

**Published:** 2009-10-05

**Authors:** Maxim A. Khasnatinov, Katarina Ustanikova, Tatiana V. Frolova, Vanda V. Pogodina, Nadezshda G. Bochkova, Ludmila S. Levina, Mirko Slovak, Maria Kazimirova, Milan Labuda, Boris Klempa, Elena Eleckova, Ernest A. Gould, Tamara S. Gritsun

**Affiliations:** 1 The University of Reading, School of Biological Sciences, Whitenights, Reading, United Kingdom; 2 Institute of Virology, Slovak Academy of Sciences, Bratislava, Slovakia; 3 Chumakov Institute of Poliomyelitis and Viral Encephalitides, Russian Academy of Sciences, Kiev Shosse, Moscow, Russia; 4 Institute of Zoology Slovak Academy of Sciences, Bratislava, Slovakia; 5 Institute of epidemiology and microbiology SC FHHR SD RAMS, Irkutsk, Russia; 6 Institute of Virology, Helmut-Ruska-Haus, Charité School of Medicine, Berlin, Germany; 7 Unité des Virus Emergents, Faculté de Médecine Timone, Marseille, France; 8 Centre for Ecology and Hydrology (CEH) Oxford, Oxford, United Kingdom; University of Pretoria, South Africa

## Abstract

*Tick-borne encephalitis virus* (TBEV) causes human epidemics across Eurasia. Clinical manifestations range from inapparent infections and fevers to fatal encephalitis but the factors that determine disease severity are currently undefined. TBEV is characteristically a hemagglutinating (HA) virus; the ability to agglutinate erythrocytes tentatively reflects virion receptor/fusion activity. However, for the past few years many atypical HA-deficient strains have been isolated from patients and also from the natural European host tick, *Ixodes persulcatus*. By analysing the sequences of HA-deficient strains we have identified 3 unique amino acid substitutions (D67G, E122G or D277A) in the envelope protein, each of which increases the net charge and hydrophobicity of the virion surface. Therefore, we genetically engineered virus mutants each containing one of these 3 substitutions; they all exhibited HA-deficiency. Unexpectedly, each genetically modified non-HA virus demonstrated increased TBEV reproduction in feeding *Ixodes ricinus*, not the recognised tick host for these strains. Moreover, virus transmission efficiency between infected and uninfected ticks co-feeding on mice was also intensified by each substitution. Retrospectively, the mutation D67G was identified in viruses isolated from patients with encephalitis. We propose that the emergence of atypical Siberian HA-deficient TBEV strains in Europe is linked to their molecular adaptation to local ticks. This process appears to be driven by the selection of single mutations that change the virion surface thus enhancing receptor/fusion function essential for TBEV entry into the unfamiliar tick species. As the consequence of this adaptive mutagenesis, some of these mutations also appear to enhance the ability of TBEV to cross the human blood-brain barrier, a likely explanation for fatal encephalitis. Future research will reveal if these emerging Siberian TBEV strains continue to disperse westwards across Europe by adaptation to the indigenous tick species and if they are associated with severe forms of TBE.

## Introduction

Tick-borne encephalitis virus (TBEV) causes up to 14,000 human cases of tick-borne encephalitis (TBE) across Eurasia annually [1], [2]. TBE outbreaks are now registered in about 30 European countries with a recorded morbidity increase of about 400% during the past 30 years [3]. TBEV is a member of the tick-borne flavivirus (TBFV) group that, together with mosquito-borne and no-known vector virus groups comprise the genus *Flavivirus* within the family *Flaviviridae*. Human pathogens within the genus *Flavivirus* include Japanese encephalitis virus, Dengue virus and Yellow fever virus that cause annual epidemics of fever, encephalitis and hemorrhagic fever in the tropics and some sub-tropical regions [4], [5].

In its natural habitat, TBEV is maintained by transmission between infected and non-infected ticks when they co-feed on small forest animals [6]–[8]. Humans are incidental hosts for ticks and may become infected by a feeding infected tick. The clinical manifestations caused by TBEV range from inapparent infections and fevers, with complete recovery of patients, to debilitating or fatal encephalitis. The proportion of fatal human infections varies widely in different regions and in different years. The factors that determine disease severity are poorly defined but correlations between viral subtype and disease severity have been described. TBEV strains are currently divided into 3 closely related subtypes, i.e. western-European (WE), Siberian (SIB) and Far Eastern (FE) [9]. FE TBEV is recognised as the most virulent pathogen with a 20–40% case fatality rate. The SIB subtype is considered less virulent (7–8% case fatality rate) but chronic disease occurs more frequently (1–3%). Western European strains are the least virulent with case fatality rates lower than 2%. However, a range of clinical manifestations, from asymptomatic to encephalitic is observed for all TBEV subtypes [1], [2] and the underlying basis for this has not yet been adequately explained.

Conventionally, each TBEV subtype has been associated with distinct geographic ranges within the Old World region of the northern hemisphere, hence the groupings Far East, Siberia and Western Europe [9]. However during recent decades the epidemiology of the TBFV appears to have been changing, with SIB TBEV becoming the dominant subtype apparently gradually replacing the WE or FE subtypes that previously appeared to monopolise many regions [10]–[16]. Moreover, the SIB subtype is being isolated more frequently from patients who develop the most severe forms of encephalitis, with the virus invading the entire brain in contrast with the more focal virus localization observed previously. Over a period of time, the most severe cases of TBE have been more frequently associated with the SIB strains than with the FE strains [17], indicating that this is not an artifact of increased surveillance. Whilst these reports are disturbing they have not as yet been addressed at the molecular virological level.

TBEV virions are spherical particles with an ∼11 kb-RNA genome embedded in a capsid that is surrounded by a lipid envelope mainly containing a virus envelope (E) glycoprotein. This E protein plays a key role in many stages of the virus life cycle; it mediates virus binding to receptors on the cell surface (adsorption) which triggers receptor-mediated endocytosis. Exposure of the endocytosed virus to the acid pH converts the native E protein dimers into fusogenic trimers [18], [19]; the latter promote fusion of virion and endosomal membranes thus releasing viral RNA into the cytoplasm. The E protein also plays the major role in inducing the host immune response and mediates hemagglutination (HA), i.e. the ability of virions to agglutinate avian erythrocytes; for decades HA has been used in routine diagnosis [20].

Whilst most strains of TBEV show HA activity, during the past 10 years atypical HA-deficient strains have been isolated with increasing frequency in Europe from both ticks and infected patients. More than 40 HA-deficient strains are now recognized and they all exhibit reduced pathogenicity for mice when compared with HA-competent strains. They are also called “antigenically deficient” (AD) strains, in contrast to the more common antigenically competent (AC) strains; the term “AD” is derived from the strict correlation between loss of HA and immunoprecipitating activities [21]. The AD-viruses were also deficient in complement-fixation and neutralization tests when analysed using either hyperimmune antisera or sera obtained from patients recovered from TBE [21]. Here, using molecular methods of analysis, we show that HA-deficiency is linked with the adaptation of SIB TBEV strains to western European *Ixodes ricinus* ticks reflecting altered, E protein-mediated, receptor/fusion functions. We also illustrate how this might result in continued westward dispersal and emergence of new highly pathogenic virus variants (see Fig. S1).

## Results

### Yar viruses are HA- deficient TBEV strains

Yar-strains of TBEV i.e. Yar71, Yar 114, Yar 46-2, and Yar 48 were isolated in the European part of Russia (Yaroslavl' region) between 1999–2001 and details for their isolation are listed in Table 1. All 4 Yar viruses had equivalent infectivities when compared with the Vasilchenko (Vs) strain of TBEV (see Methods) used as the positive control virus (Table 1) and produced comparable concentrations of E protein when analysed by Western blot (data not shown). However, they were completely negative in HA tests over a range of pH 5.75–7, regardless of whether they were prepared in newborn mice or in PS cells. The control Vs virus and pGGVs virus, recovered from the infectious clone (see Methods), produced high positive HA titres (1∶640-1∶1280) at pH 6.2 and this was therefore the pH of choice for all subsequent tests (Table 1). Thus, the 4 Yar isolates can be defined as HA- or AD-deficient in common with the other 40 strains that have been isolated in Europe and were also identified as HA- and AD-deficient [21].

**Table 1:** TBEV strains used in this study.

Strain	Year of isolation	Source of isolation	Passage history	Accession No	HA test (titers)	Infectivity in PS cells on 72 h pi (PFU/ml)
Vs	1961	Patient blood	Unknown	L40361	1∶1280	2−8×10^6^
Yar 71	1999	*I. persulcatus*	7 in PS	EU444077	0	2−8×10^6^
Yar114	2001	*I. persulcatus*	4 in PS and 2 in mice	EU444078	0	2−8×10^6^
Yar 46-2	2001	Patient CNS	5 in PS and 2 in mice	EU444079	0	2−8×10^6^
Yar 48	2000	*I. persulcatus*	5 in PS	EU444080	0	2−8×10^6^
pGGVs	2007	Infectious clone	1 in PS		1∶640	2−8×10^6^
IC-D67G	2007	Infectious clone	1 in PS		0	2−8×10^6^
IC-E122G	2007	Infectious clone	1 in PS		0	2−8×10^6^
IC-D277A	2007	Infectious clone	1 in PS		0	2−8×10^6^
IC-T175N	2007	Infectious clone	1 in PS		1∶640	2−8×10^6^

### Yar- viruses belong to the “Baltic” group of the Siberian TBEV subtype

**Figure 1:**
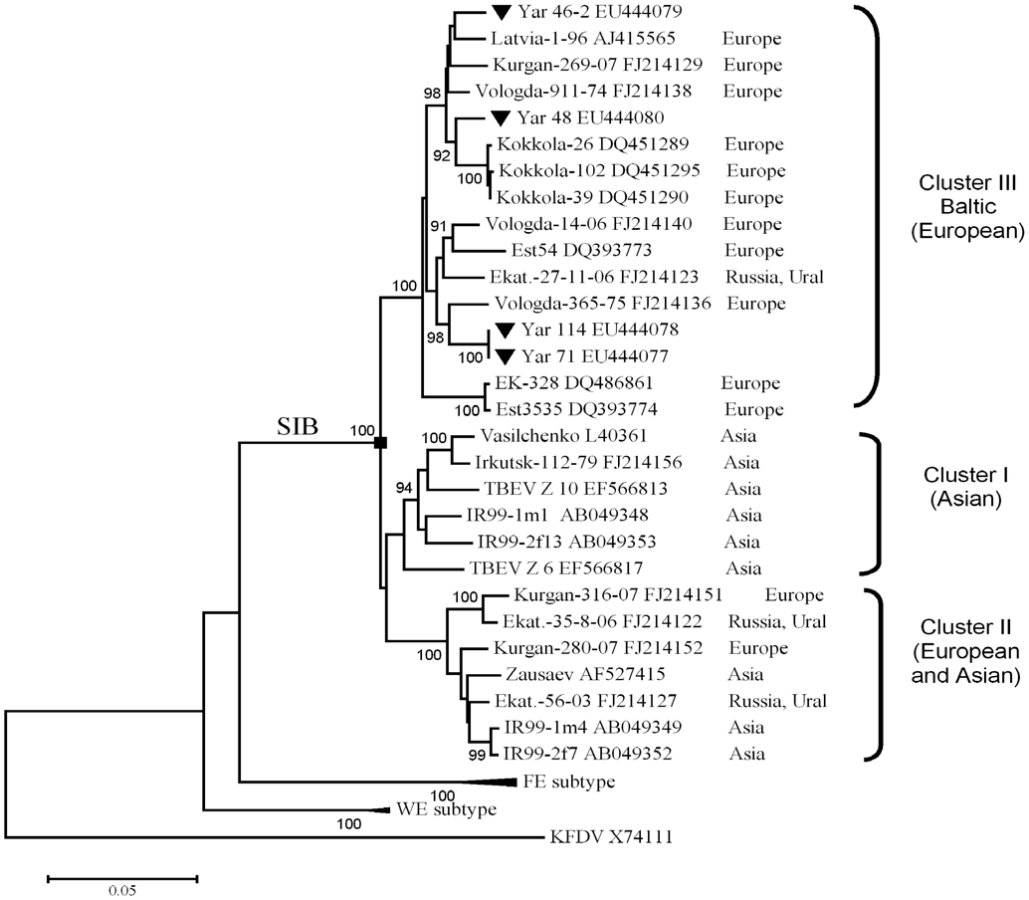
Phylogenetic analysis of Yar strains. MEGA version 4 [43] was used to align E genes (between nucleotide positions 1114–2223 of Vs virus genome) of SIB TBEV strains (accession numbers are specified). Tree topology was reconstructed by Neighbor-Joining. The Tamura-Nei model was used for estimation of evolutionary distances [44]. Bootstraps were based on 1000 replications; values below 90% are hidden. The scale bar shows the number of nucleotide substitutions per site. Geographic origins of SIB strains and clusters I, II and III are shown on the right hand side of the tree. Yar strains are highlighted using triangles. KFDV was used as the outgroup.

Phylogenetic analyses based on a 1110-bp fragment of the E gene (positions 1114–2224 in Vs virus L40361) showed that the Yar-viruses belong to the SIB subtype of TBEV [9],[22]–[26], which includes the control Vs virus (see Methods). Fig. 1 illustrates the overall branching pattern in agreement with previously published results [24], [25]. Three Siberian sub-clusters I, II and III were clearly identifiable and supported by high bootstrap values. Yar-viruses were grouped with strains of sub-cluster III designated “Baltic” [27], isolated only in Europe, in contrast to strains of clusters I and II that were found across Europe and Asia. Separate phylogenetic analyses based on the C, prM, E and NS1 genes produced trees that were congruent with the one presented (data not shown).

### Hemagglutination deficiency results from single amino acid substitutions on the virion surface

**Figure 2:**
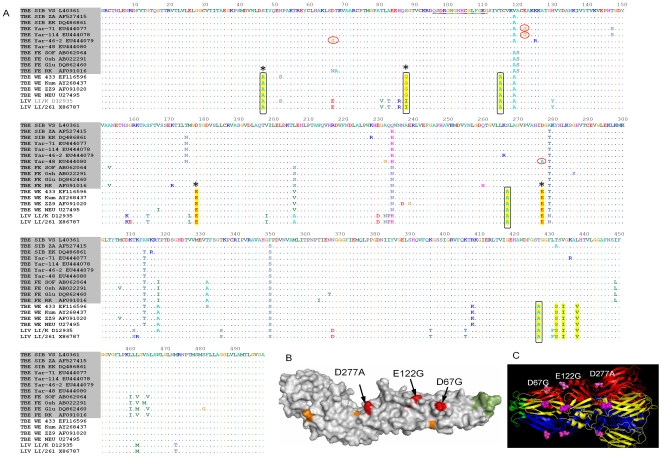
Identification of HA-disabling mutations. (A). Abbreviated comparative alignment of TBEV E protein sequences (complete version is available on request). TBEV strains are specified by subtype (FE-, SIB- or WE-subtypes) and GenBank accession numbers. HA-disabling mutations are encircled. The “tick-specific” amino acids that differentiate *I. persulcatus*-transmitted viruses (shadowed) from the *I. ricinus*-transmitted viruses are highlighted in yellow; those that increase hydrophobicity are boxed and surface-faced amino acids are marked with asterisks (*). The fusion peptide is underlined. (B) Mapping of HA-disabling residues onto native dimeric conformation of E protein crystal structure [45] (1SVB.pdb); the monomer is shown as it lies on the virion membrane. The “persulcatus” and “ricinus” residues are highlighted in orange; fusion peptide is in green and HA-deficient amino acids (arrows) are red. The position of Yar substitution D277A (red coloured) coincides with the position of “ricinus/persulcatus” substitution (orange colour is masked). (C) Residues 67, 122 and 277 (purple spheres) are mapped onto the E protein in trimeric post-fusion conformation (1URZ.pdb) [18]. The fusion peptide is highlighted in green and can be seen protruding out of the virion membrane towards the endosomal membrane. Different subunits of the trimer are coloured in red, blue and yellow.

To identify amino acid(s) responsible for the loss of HA-activity, we sequenced Yar viruses and aligned them with 290 available TBEV E sequences (Fig. 2A). One amino acid 175N in the E protein was common to all HA-deficient Yar-viruses, in contrast with the highly conserved 175T. Therefore, the substitution T for N at amino acid position 175 was introduced into a TBEV infectious clone (IC) designated pGGVs [28], [29] to generate mutant virus IC-T175N that produced HA titres similar to parent Vs virus and also control pGGVs virus rescued from the infectious clone (Table 1). Since no other common amino acids were identified that distinguished Yar-viruses from the other strains, we hypothesised that individual non-shared amino acid substitutions might be responsible for the HA-deficient phenotype. The alignment in Fig. 2A revealed 3 non-conserved mutations, each of which was unique to one of the Yar strains, i.e. D67G (Yar 46-2), E122G (Yar 71 and Yar 114) and D277A (Yar 48). When compared with the parent pGGVs virus, each of these mutations increased net charge and hydrophobicity of the E protein and was surface orientated, mapping on the most protruding loops of the E protein in its native dimeric conformation (Fig. 2B).

The loss of HA activity in TBEV has only previously been reported in relation to selective adaptation of TBEV to ticks. One substitution, E87K was generated during the propagation of a WE strain to *I. ricinus* ticks [30] and two substitutions, E122G and T426I respectively, followed a few SIB TBEV passages in *H. marginatum* ticks [31]. In support of our observations, the E protein surface in these independently reported HA deficient viruses, is predicted to be of either positive or neutral charge, as we have described for the substitutions, D67G, E122G and D277A respectively.

Whilst the molecular details of interactions between virions and erythrocytes remain unknown it has been suggested that HA activity might be mediated by the trimeric E protein in its post-fusion conformation rather than by native E dimers [32]. Five of these trimers form a fusion pore that enables fusion between the viral and cellular endosomal membrane thus releasing the viral RNA into the cellular cytoplasm. Therefore we mapped the chimaeric Yar-simulated mutants onto the crystal structure of the trimeric (post-fusion) conformation of the E protein [18]. This demonstrated that the 3 Yar-virus mutations are located along the most protruding parts of the lateral surface of the trimer (Fig. 2C). Therefore they are likely to be able to make direct contact with the erythrocyte surface and/or with each other.

**Figure 3:**
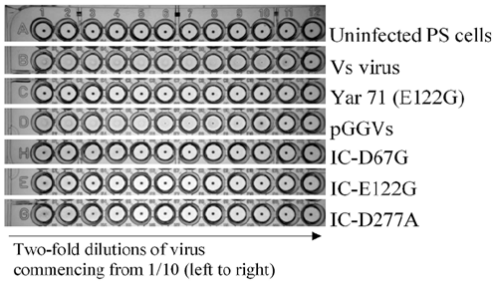
Effect of Yar-virus simulated mutations on HA activity of TBEV. The engineered viruses IC-D67G, IC-E122G, IC-D277A and pGGVs (1−4×10^7^ PFU/well) were tested for HA using chicken erythrocytes at pH 6.2.

Thus, we hypothesised that each of these three amino acid substitutions could individually abolish HA deficiency in TBEV. To test this hypothesis experimentally we used a TBEV infectious clone to engineer three mutant viruses IC-D67G, IC-E122G and IC-D277A (See Methods) that simulate the wild-type viruses Yar 46-2, Yar 71/Yar 114 and Yar 48 (Table 1). The introduction of any one of the three mutations into the parent HA positive pGGVs virus rendered it HA negative (Fig. 3).

### The HA-deficient engineered mutants show slower growth rates in mammalian cells

**Figure 4:**
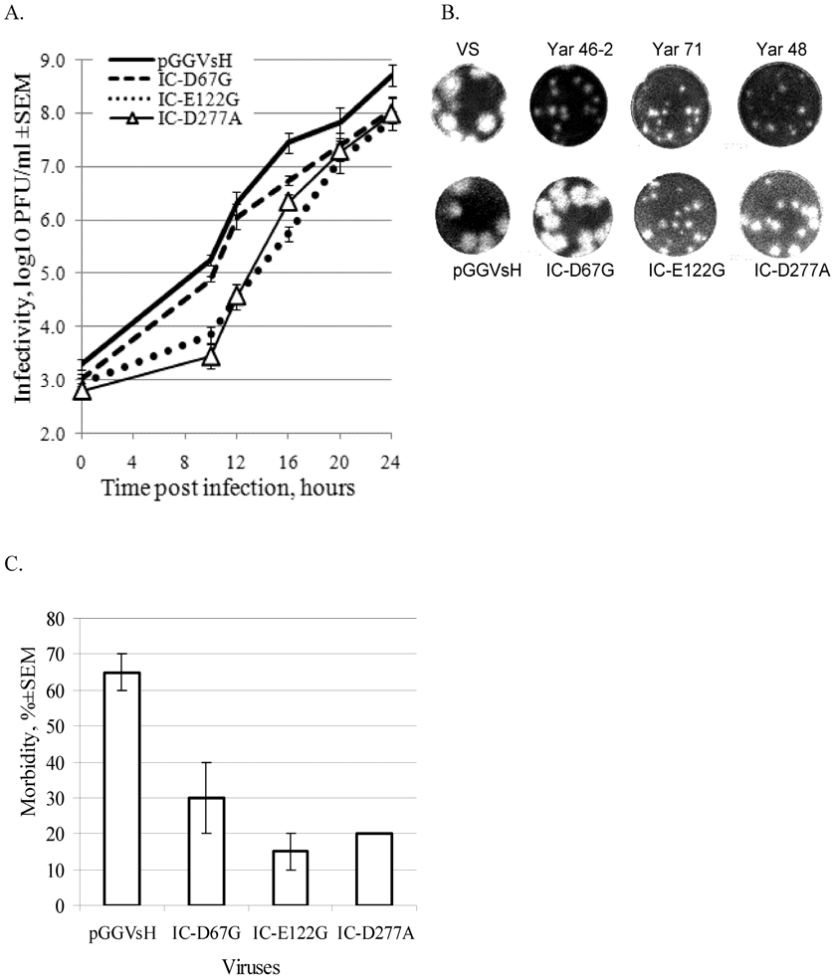
Effect of HA-disabling mutations on growth characteristics of TBEV in mammalian models. (A) Confluent monolayers of PS cells were infected with IC-D67G, IC-E122G, IC-D277A or pGGVsH virus at an estimated moi of 1 PFU/cell and virus yield in cell culture medium was determined at different time points post-infection by plaque assay. (B) Plaque morphology of specified Yar viruses and engineered mutants in PS cell monolayers. (C) Adult mice were inoculated intraperitoneally with 2000 PFU of IC-D67G, IC-E122G, IC-D277A or pGGVsH followed by monitoring of the morbidity rate (neuroinvasiveness test).

All three engineered mutants demonstrated delayed growth in PS cells, most visible during the first 24 hours (Fig. 4). Nevertheless, they all subsequently reached titres similar to pGGVs HA-positive virus by day 3 (Table 1). Although, mutant IC-D67G exhibited better growth characteristics than IC-E122G and IC-D277A it was reproducibly slightly lower than the control pGGVs virus.

All 3 natural HA-negative isolates (Yar71, Yar 46-2, and Yar 48) displayed small-plaque phenotype (1 mm, Fig. 4B), in comparison with control Vs strain which produced much larger plaques (3 mm) but only two corresponding mutations, E122G and D277A caused plaque size reduction in the engineered viruses (0.7 to 1.5 mm as shown in Fig. 4B). Clearly, as yet unidentified additional mutations contributed to the small plaque phenotype of Yar 46-2 (D67G). Similarly Yar 48 virus formed smaller plaques (1 mm) than the corresponding IC-D277A mutant (1.5 mm) demonstrating that HA activity and plaque phenotype are not always determined by the same amino acid.

Vs virus is different from many other laboratory-maintained TBEV strains; in cell culture it develops cytopathic effect (cpe) relatively slowly [22]. All tested mutants showed no increase or decrease of cpe in comparison with the control infectious clone pGGVs, even at high multiplicity of infection (10 PFU/cell).

### HA-deficient mutants show reduced TBEV neuroinvasiveness for mice

After ip inoculation, the control virus pGGVs produced a relatively high morbidity rate (65%) in correspondence with previous results [29]. In contrast, all three engineered virus mutants exhibited lower neuroinvasiveness as determined by morbidity rates (Fig. 4C). Mice observed for 21 days following ip inoculation with IC-D67G produced antibodies against TBEV, indicating that they had been infected (data not shown).

### HA-deficiency correlates with increased TBEV reproduction in feeding ticks and tick-to-tick transmission efficiency

**Figure 5:**
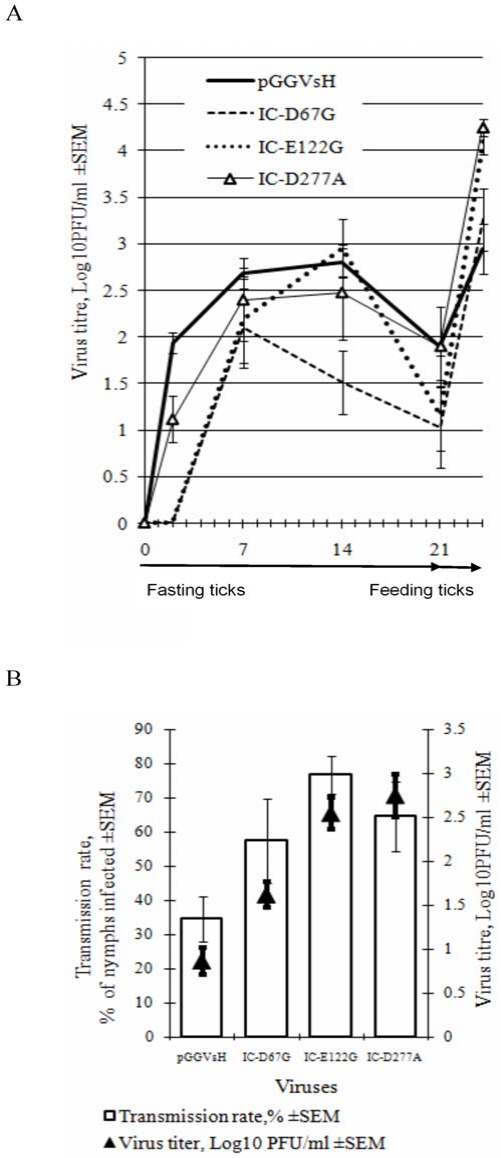
Effect of HA-disabling mutations on TBEV replication in ticks. (A) Adult unfed *I. ricinus* females were infected with IC-D67G, IC-E122G IC-D277A or pGGVsH virus (day 0) and virus yield (lgPFU/ml; solid bars) was determined after incubation of infected fasting ticks for 2, 7, 14 and 21 days and also following the transfer of 21-day fasting ticks to feed on the mice for 3 days. (B) For tick-to-tick transmission, adult infected ticks were placed on mice in close proximity to uninfected nymphs and allowed to co-feed for 3 days. Tick-to-tick transmission rate (clear bars) is expressed as the proportion of infected nymphs to the total number of nymphs. Black triangles show the average virus titres in individually infected recipient nymphs as determined by plaque assay.

Studies on the reproduction and tick-to-tick transmission efficiency of IC-D67G, IC-E122G and IC-D277A were carried out using a novel tick/mouse laboratory model initially developed by Labuda et al [7]. In the first set of experiments, 30 ticks were infected by injection in the leg with each engineered virus as described in Methods and TBEV titres in salivary glands were measured in each of 6 ticks at each time point, i.e. on 2^nd^, 7^th^, 14^th^ and 21^st^ day following infection. The reproduction characteristics of IC-E122G and IC-D277A in fasting ticks were similar to those of control pGGVs virus whereas the titres of IC-D67G were significantly lower (Fig. 5A).

On the 21^st^ day post-infection, fasting infected ticks were allowed to feed on mice and virus titres were measured in each of 6 individual ticks. This analysis was employed for each engineered virus, 3 days after feeding. Fig. 4A shows that infectivity of IC-E122G had increased approximately 1000-fold and IC-D277A and IC-D67G had increased approximately 300-fold, whereas for control pGGVs virus the increase of virus titre was about 10-fold. Since 6 ticks were used for each tested virus for each time-point, the differences in titres between different virus mutants were statistically significant, based on Student t-tests (p<0.05).

In the second set of experiments, the efficiency of virus transmission from infected adult female ticks to uninfected nymphs during co-feeding on mice (tick-to-tick transmission; see Methods) was evaluated by estimating the proportion of infected nymphs. In addition, the titres of virus in each recipient nymph were estimated in a plaque assay. The results show in each case, that HA deficiency directly correlated with increased TBEV titres in nymphs, following feeding and also tick-to-tick transmission efficiency (Fig. 5B).

### Analysis of non-conserved amino acid substitutions on the virion surface

We analysed 290 TBEV E protein sequences for the presence of amino acid substitutions similar to those that resulted in the loss of HA in TBEV, i.e. acidic (positively charged) residues that were replaced by hydrophobic and/or neutral amino acids (i.e. glycine) and localized on the virion envelope protein surface (Table 2). In total, 5.8% (including Yar-viruses) of the strains exhibited similar mutations. Strains with potentially increased charge and/or surface hydrophobicity were identified in all three TBEV subtypes, i.e. FE, SIB and WE; they were isolated from different geographical regions and a variety of hosts including ticks, rodents and humans.

**Table 2:** The amino acid substitutions of E protein of TBEV strains (290 viruses were analyzed) that resulted in the change of net charge and/or hydrophobicity of virion surface E-gene.

Mutation	TBEV strain	Accession No	TBEV subtype	Source of isolation	Region of isolation	Clinical manifestations (if known)
D67G	Est2546	DQ393779	FE	Field mouse	Estonia	
D67G	T-blood	AF091019	FE	TBE patient	Ural	Meningoencephalitis
D67G	Ural-Nina	FJ214119	FE	TBE patient	Ural	Meningoencephalitis
D67G	Ural-P	FJ214118	FE	TBE patient	Ural	
D67G	Ural-B	FJ214117	FE	TBE patient	Ural	
D67G	Ural-A	FJ214115	FE	TBE patient	Ural	
D67G	Volkhov-K	FJ214114	FE	TBE patient	Europe	Chronic encephalitis
D67N	RK 1424	AF091016	FE	*I. persulcatus*	Latvia	
E84K	1486	EF469755	SIB	*I. persulcatus*	Siberia	
E84G	4387/7	X76608	WE	*I .ricinus*	Slovakia	
E122G	DXAL5	AY178833	FE	Not specified	China	
E122G	KrM219	DQ988684	WE	Rodents	Korea	
E155G	272-75	AF231806	SIB	Vole	Siberia	
E170G	Koltsovo-29	AF540032	FE	TBE patient	Siberia	
D203G	DXAL-12	EU089977	FE	Not specified	China	
D203G	DXAL-13	EU089976	FE	Not specified	China	
D203G	DXAL-21	EU089980	FE	Not specified	China	

Notably, the substitution D67G was detected in 7 viruses isolated only from mammalian hosts and human patients. No other correlation between isolation source, geography or virus subtype specificity was observed. A significant number of TBEV E proteins have been sequenced partially (227 viruses of 290 available from GenBank), therefore it could not be excluded that TBEV strains with increased surface charge/hydrophobicity are quite common in nature. Indeed among 63 completely sequenced E proteins those with increased charge/hydrophobicity on the virion surface comprise ∼20% (including Yar viruses).

We also compared E proteins of TBEV strains isolated from *I. ricinus* (WE-strains) with those isolated from *I. persulcatus* (FE- and SIB-strains), to identify group amino acids that might be involved in TBEV adaptation to these two different tick species (Fig. 2A). Ten amino acid differences were revealed that have previously been localised in hypervariable clusters of the envelope protein [33] and five showed overall increased E protein hydrophobicity in the *I. ricinus*–transmitted WE-strains in contrast with FE-and SIB-strains (Fig. 2A). Four of these ten substitutions were localised on the virion surface, with two (S47A and S88G) increasing the surface hydrophobicity of “ricinus” strains and two (D178E and D277E) being conserved (Fig. 2B). Remarkably, the position of the “tick”-specific (i.e. *I. ricinus* or *I. persulcatus*) D277E amino acid substitution overlapped with the D277A substitution of Yar 48 (Figs. 2A and 2B). Two substitutions that also increased the hydrophobicity of strains adapted to *I. ricinus* were localised either inside the E protein (T115A) or on the membrane-oriented side (S267A), i.e. under the virion surface. Four substitutions (T427A, T431S, V433I and L437V) were located in the transmembrane domain of the E protein, with one (T427A) more hydrophobic for “ricinus” strains. Louping ill virus (LIV), a TBEV-related virus which is transmitted by *I. ricinus* in the UK, also showed the same more hydrophobic pattern and localisation of “tick-specific” amino acids as the *I. ricinus*-transmitted WE TBEV strains (Fig. 2A).

## Discussion

TBE is currently reported in more than 30 countries of Eurasia and causes significant outbreaks of encephalitis in 16 countries, including 13 EU Member States (Austria, the Czech Republic, Estonia, Finland, Germany, Greece, Hungary, Latvia, Lithuania, Poland, Slovak Republic, Slovenia, Sweden) and three non-EU Member States (Norway, Russia and Switzerland) [3]. The clinical manifestations of TBE in endemic regions vary widely, from inapparent and febrile infections, with recovery of patients, to debilitating or fatal encephalitis, even in some vaccinated individuals [1], [2] and no adequate explanations have as yet been produced.

Here, we have investigated the molecular mechanisms and epidemiological implications of the emergence of unusual TBEV strains originally identified as HA- or AD-deficient [21]. In contrast with most TBEV strains, these novel viruses fail to agglutinate avian erythrocytes, show reduced antigenic characteristics and replicate relatively poorly in mammalian cells. We demonstrated that these strains display increased hydrophobicity and positive charge on their virion surface. By engineering genetically modified viruses we proved that these distinct surface characteristics, including HA-deficiency, are caused by any one of three single amino acid substitutions D67G, E122G or D277A in the E protein. These mutations significantly increased virus reproduction in feeding ticks and increased the efficiency of tick-to-tick virus transmission when infected and uninfected ticks co-fed on the same animal. Thus mutations leading to HA- and AD-deficiency are directly associated with selection for enhanced virus transmission between ticks, a process that facilitates virus survival in the natural habitat [7].

Although *I. ricinus* ticks can be routinely maintained in laboratories, SIB TBEV is normally associated with transmission by *I. persulcatus*, which is not as readily available for laboratory experiments. Nevertheless, WE TBEV strains show ∼100% transmission efficiency in their natural tick vector *I. ricinus* (manuscript in preparation). Thus, logically, Yar viruses (i.e. SIB TBEV strains) should be transmitted efficiently in their natural vector *I. persulcatus*. Geographically, *I. ricinus* and *I. persulcatus* overlap in Europe and numerous reports describe the isolation of Siberian strains from *I. ricinus* which is now recognised as a second vector for SIB TBEVs [11], [34]. We therefore propose that the driving force behind the westward dispersal of these HA-deficient strains is their adaptation to newly-encountered European *I. ricinus* ticks. This hypothesis is also supported by our analysis of E protein comparative alignments between FE, SIB and WE subtypes that revealed more hydrophobic “ricinus” amino acid patterns, compared with “persulcatus”, in correspondence with tick preference of WE or FE/SIB strains respectively. *Louping ill virus*, which is transmitted by *I. ricinus* in the UK, shares this “more hydrophobic” pattern with the WE TBEVs.

For many viruses, HA-activity is recognised as a reflection of receptor [35]–[37] or low-pH dependent fusion activity [38], [39]. It was suggested that HA activity of flaviviruses is mediated by fusion activity of the E protein [32] because it is optimal at pH 6.2 which promotes conversion of native E protein dimers into fusion-active trimers [18], [19]. This implies that the Yar mutations identified herein destabilise trimer-trimer contacts or contact between trimers and erythrocyte membranes, thus preventing HA activity. Alternatively, these mutations may impact on both E protein functions, ie virus adsorption to the cell surface and pH-dependent fusion of virions with endosomal membranes.

Therefore, depending on charge and hydrophobicity, tick cell receptors may restrict “easy” entry of WE-strains into *I. persulcatus* or SIB-strains into *I. ricinus*. Clearly these barriers to infection are not absolute since Vs virus (SIB TBEV) has a limited capacity to replicate in *I. ricinus* (Fig. 5). Phylogenetic analysis also supports our hypothesis; it was proposed that WE-strains diverged from ancestral FE- and SIB lineages [40], implying that the emergent WE subtype adapted to *I. ricinus* from *I. persulcatus* by evolving a more hydrophobic E protein (Fig. 2). The coincidence of increased hydrophobicity of the virion surface for *I. ricinus-*adapted WE-strains and SIB Yar viruses presumably reflects a similar molecular requirement for different viruses to adapt to the same host. Alternatively, the emergence of the atypical Yar viruses may result from adaptation of TBEV to both tick species. Indeed SIB TBEV strains have been isolated from both *I. ricinus* and *I. persulcatus* on numerous occasions [11], [34] and regular switching between them cannot be excluded. These data might explain the apparently increasing dissemination of SIB TBEV in Europe; in a few decades this virus could reach more western territories, possibly even the UK where *I. ricinus* is the vector for the *Louping ill virus* that is closely related to TBEV.

The mutant IC-D67G was distinct from other HA-deficient TBEV strains since there was no obvious correlation between loss of HA activity and significantly reduced growth in mammalian cells. The Yar 71 virus, with the corresponding D67G mutation was isolated from a fatally infected individual (Table 1) and ominously, similar substitutions have also been detected in other TBEV isolates from hospitalised patients with encephalitis (Table 2). It is possible that due to altered surface charge and hydrophobicity, strains with D67G might be more able to penetrate the human blood-brain barrier (neuroinvasiveness) or more rapidly spread between human neurones, with no correlation to reduced mouse neuroinvasiveness. This would explain the recently discovered association of the most severe form of human encephalitis with the SIB strains [17].

The molecular basis of antigenic deficiency of Yar viruses has not been elucidated but it might be related to the increased incidence of the most severe forms of TBE having been associated with SIB subtype [17]. Clearly more studies are required before we can understand, at the molecular level, the implications of the phenomenon of HA- and AD-deficiency in terms of the development of TBE from fever to encephalitis as an interplay between virus neuroinvasiveness and ability to evade the immune response.

Thus, the emergence of HA-deficient TBEV mediated by adaptation to different tick species might represent a mechanism for the westward dissemination of SIB TBEV, increased TBE incidence in Europe, and might also be the reason for encephalitis in humans (see Fig. S1). To confirm and develop these ideas, future research should focus on large-scale genomics and transmission studies of TBEV isolates recovered from patients and ticks.

## Methods

### Viruses, cells, ticks and antisera

Porcine embryo kidney cells (PS) were used to produce TBEV stocks, to recover mutant viruses, for plaque assay and studies of cytopathogenicity. Yar-strains of TBEV i.e. Yar71, Yar 114, Yar 46-2, and Yar 48 were isolated in the European part of Russia (Yaroslavl' region) between 1999–2001 (Table 1) and stored as 10% mouse brain suspensions. SIB TBEV strain Vasilchenko (Vs) and its infectious clone (pGGVs) used as control viruses have been described previously [22], [28]. *I. ricinus* ticks were bred in the Institute of Zoology, Slovak Academy of Science, Bratislava [7].

### RNA extraction, reverse transcription (RT), PCR and sequencing of Yar viruses

The RNA of each Yar virus was extracted from 200 µl of 10% infected mouse brain suspension or infected PS cell supernatant using Total RNA Isolation System (PROMEGA). The RT-PCR was used to amplify the 5′-C-prM-E gene region of Yar viruses as described [28], [41]. PCR products were directly sequenced using a Taq BigDye Terminator v3.1 Cycle Sequencing Kit (Applied Biosystems). Sequences of the C-prM-E region of Yar-viruses were deposited in GenBank with accession numbers EU444077, EU444078, EU444079 and EU444080 (Table 1).

### Phylogenetic analysis

Nucleotide and deduced amino acid sequences were aligned using program package BioEdit [42]. Phylogenetic analyses were conducted using MEGA4 [43]. Tree topology was reconstructed by the Neighbor-Joining method and the Tamura-Nei model was used for estimation of genetic distances [44]. The reliability of the tree was evaluated by bootstrapping based on 1000 replications.

### Mapping of amino acid substitutions on the crystal structure of the TBEV E protein

The unique amino acid substitutions in the E protein of each Yar virus were mapped onto the crystal structure of the native dimeric [45] or low-pH induced trimeric [18] conformation (1SVB.pdb and 1URZ.pdb respectively ) using PyMOL program (DeLano, W.L. The PyMOL Molecular Graphics System (2002) on World Wide Web http://www.pymol.org).

### Cytopathic effect (cpe), plaque assay and virus growth cycles in PS cells

The routine protocols for cpe, plaque assay and growth curve experiments were described in detail previously [25], [28], [29], [41]. Briefly, for cpe and growth curve experiments PS cells in 24-well plates were infected with viruses at a multiplicity of infection (moi) of 1 PFU/cell, in four replicates. To estimate cpe, the inoculum was replaced with RPMI medium after virus adsorption for 1 h at 37C. Infected cell monolayers were initially examined by microscopy and then stained with 0.1%naphthalene black at 24, 48, 72, 96 or 120 h post-infection for further examination of the extent of cpe. For plaque assay, original virus stocks were 10-fold serially diluted and after 1-h of virus adsorption at 37°C infected monolayers were overlaid with 1% SeaPlaque Agarose (Cambrex, USA). After incubation at 37°C for 5 days monolayers were fixed with 10% formol saline and stained with 0.1% naphthalene black. For virus growth curve experiments, after virus adsorption for 1-h at 37°C, monolayers were washed 5 times with serum-free RPMI medium and overlaid with 1 ml of medium containing 2% FCS. The supernatant medium from infected cells was collected at 2, 10, 12, 16, 20 and 24 hours pi and frozen at −70°C. The titres of infectious virus were determined by plaque assay.

### Hemagglutination assay

PS cells were infected at a moi of 0.1 PFU/cell in 500 ml culture flasks and infectious supernatant medium was collected at day 5pi. The TBEV virions with estimated initial virus titers of 2−8×10^6^ PFU/ml were concentrated 100 times using 7% polyethylene glycol (PEG) in the presence of 2.4% NaCl overnight at 4°C and precipitated by centrifugation at 8000 rpm for 3 h. The resulting pellet was resuspended in 500 µl of PBS. For routine hemagglutination test [20], [46], 50 µl of the concentrated virus sample (10^8^ PFU/ml) were placed in the first well of 96-well plates and diluted two-fold in borate buffer (pH 9.0). Then 100 µl of a suspension of 0.5% newborn chick erythrocytes was added to each well. Results are recorded as the reciprocal of the maximum virus dilution that produced agglutination.

### Introduction of mutations into the infectious clone

**Figure 6:**
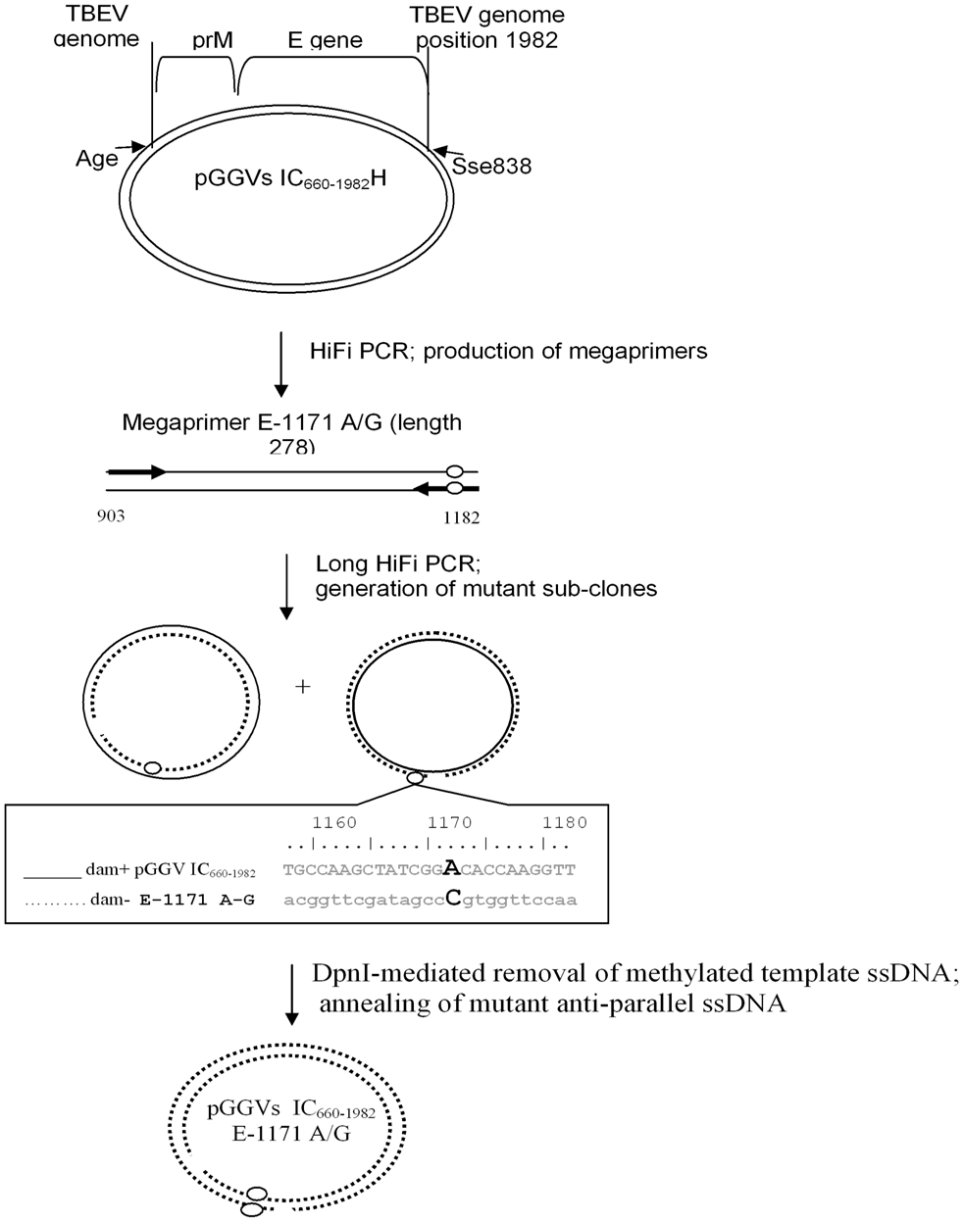
Mutagenesis of the infectious clone of TBEV. The plasmid pGGVs_660–1982_H [29] that contains the partial PrM-E gene fragment between nucleotides 660–1882 of the Vs virus genome was used as a template in PCR to synthesize megaprimers E-1171 A/G (genome positions and lengths are specified). Primers used to produce megaprimers, with targeted mutations (circles) are represented by thick arrows. Subsequently the megaprimer without the other pair of primers was used to amplify plasmid pGGVs_660–1982_H (solid circular line). The produced linear newly-synthesized complementary ssDNA molecules (nicked circular dotted line) with acquired mutations were annealed during the last step of PCR, randomly producing twice-nicked circular DNA. Parent Dam+ methylated DNA of the pGGVs_660–1982_H was removed by DpnI endonuclease digestion to facilitate clone selection.

The ligation *in vitro* of two overlapping plasmids, pGGVs_660–1982_H and pGGVs_660–1982del_ produces full-length infectious clone of TBEV strain Vs [28], [29]. The megaprimer-mediated domain swapping mutagenesis technique [47] was utilized to introduce point mutations in pGGVs_660–1982H_ (Fig. 6). For the Hi-Fi PCR, a pair of primers was used to amplify regions of about 200 nucleotides (megaprimer); one primer contained the appropriate point mutation within the E gene. Subsequently, the megaprimer was used to amplify the pGGVs_660–1982_H template in 15 cycles of circular PCR at 95°C for 30 sec, at 60°C for 30 sec and at 72°C for 5 min. Each cycle of the PCR produced nicked dsDNA molecules, with nascent (circular) DNA strand originating from template bacterial dsDNA and the other one (nicked) from newly amplified PCR product. Following 15 cycles, the accumulation of amplified linear complementary ssDNA strands resulted in the formation of annealed circular (twice nicked) molecules. To facilitate screening, the dam-methylated bacterial (template) DNA was digested with 40 U of DpnI (New England Biolabs) at 37°C for 1 h. Following this, PCR products were electroporated into AbleK bacterial cells (Stratagene) and selected clones were completely sequenced.

### Recovery of mutant viruses

To recover engineered viruses, the mutated plasmids, constructed on the basis of pGGVs_660–1982_H, were ligated with pGGVs_660–1982del_ to restore the full-length cDNA of TBEV as described previously [29]. Each mutant full-length clone was subsequently linearised by Sma *I* and used for SP6 transcription to produce full-length RNA. The SP6-transcribed RNA was transfected into PS cells using Lipofectin reagent (Invitrogen) according to the manufacturer's protocols. Infectious supernatant medium was collected on day 5 pi. The presence of virus in infected cells was confirmed by immunofluorescence microscopy using monoclonal antibodies specific for flavivirus E proteins [46] and by RT-PCR. The entire E-gene was sequenced to ensure no additional substitutions appeared during the genetic manipulation or initial virus replication in PS cells.

### TBEV replication in ticks and co-feeding transmission

Unfed adult females of *I. ricinus* ticks were inoculated with virus under a stereo zoom microscope (Wild M 400, Wild Heerbrugg AG, Switzerland) into the coaxial plate of the second pair of legs using a digital microinjector TM system (MINJ-D-CE; Tritech Research, Inc.; USA). Clean nitrogen served as a gas source to produce an injection pressure of 20 psi ( = app. 1.38bar). The injection interval was set to 1.0 sec. Hollow glass needles with a microscopically fine tip were prepared using a P-30 Micropipette puller (Sutter Instrument Company, USA).

To investigate virus reproduction in fasting ticks, groups of 45 female ticks were infected with 500 PFU/tick of one TBEV strain. Infected ticks were incubated at room temperature (24±4°C) and 85–90% RH in a desiccator for 21 days. At 2, 7, 14 and 21 days pi salivary glands of 6 ticks were dissected, individually homogenized and the concentration of infectious virus was estimated by plaque titration.

Virus tick-to-tick transmission experiments were carried out essentially as described previously [6]–[8]. Two of 45 infected adult female ticks were allowed to feed simultaneously on Balb/C mice for 3 days with 15 uninfected *I. ricinus* nymphs that were attached in close proximity (1–1.5 cm) to the feeding donor females. Surviving nymphs and salivary glands of donor females were used to determine the titres of infectious virus using plaque assays. The co-feeding transmission rate was estimated as the proportion of nymphs (%) that became infected.

Experimental animal procedures were performed in accordance with the guidelines for care and maintenance of animals (Act of the Government of the Slovak Republic 2003 regulating the use of experimental animals). All animal experiments were approved by the State Veterinary and Food Administration of the Slovak Republic (permission numbers 12284/03-220 and 2362/06-221). The ethical permission to carry out the work with mice was obtained from the Ethical Review Committee of the Institute of Virology, Slovak Academy of Sciences”.

### Neuroinvasiveness tests in mice

Ten adult ICR mice were inoculated intraperitoneally (ip) with 2000 PFU/mouse. Mice were observed for 21 days and morbidity rate was estimated as the proportion of animals that showed clinical symptoms that included hind-leg paralysis. Sick and healthy mice were tested for the presence of anti-TBEV antibodies by HA-inhibition test [20].

### Statistical analysis

Statistical analysis was performed on the data obtained from virus replication studies in PS cells and ticks, neutralization and neuroinvasiveness test using EXCEL and SigmaPlot 11 software (Systat Software Inc., USA). Standard errors of mean (SEM) were estimated for each dataset. Between-groups comparisons were performed using unpaired, two-tailed Student's t-test. Values of p<0.05 were considered as significant.

## Supporting Information

Figure S1 Cartoon illustrating the molecular mechanisms of emergence of new strains of TBEV with possible pathogenic characteristics for humans.
(10.14 MB TIF)
