# Supplementary figures and images for: Non-Hemagglutinating Flaviviruses: Molecular Mechanisms for the Emergence of New Strains via Adaptation to European Ticks
Source: PLoS One. 2009 Oct 5;4(10):e7295. doi: 10.1371/journal.pone.0007295 (PMC2750751; doi:10.1371/journal.pone.0007295)

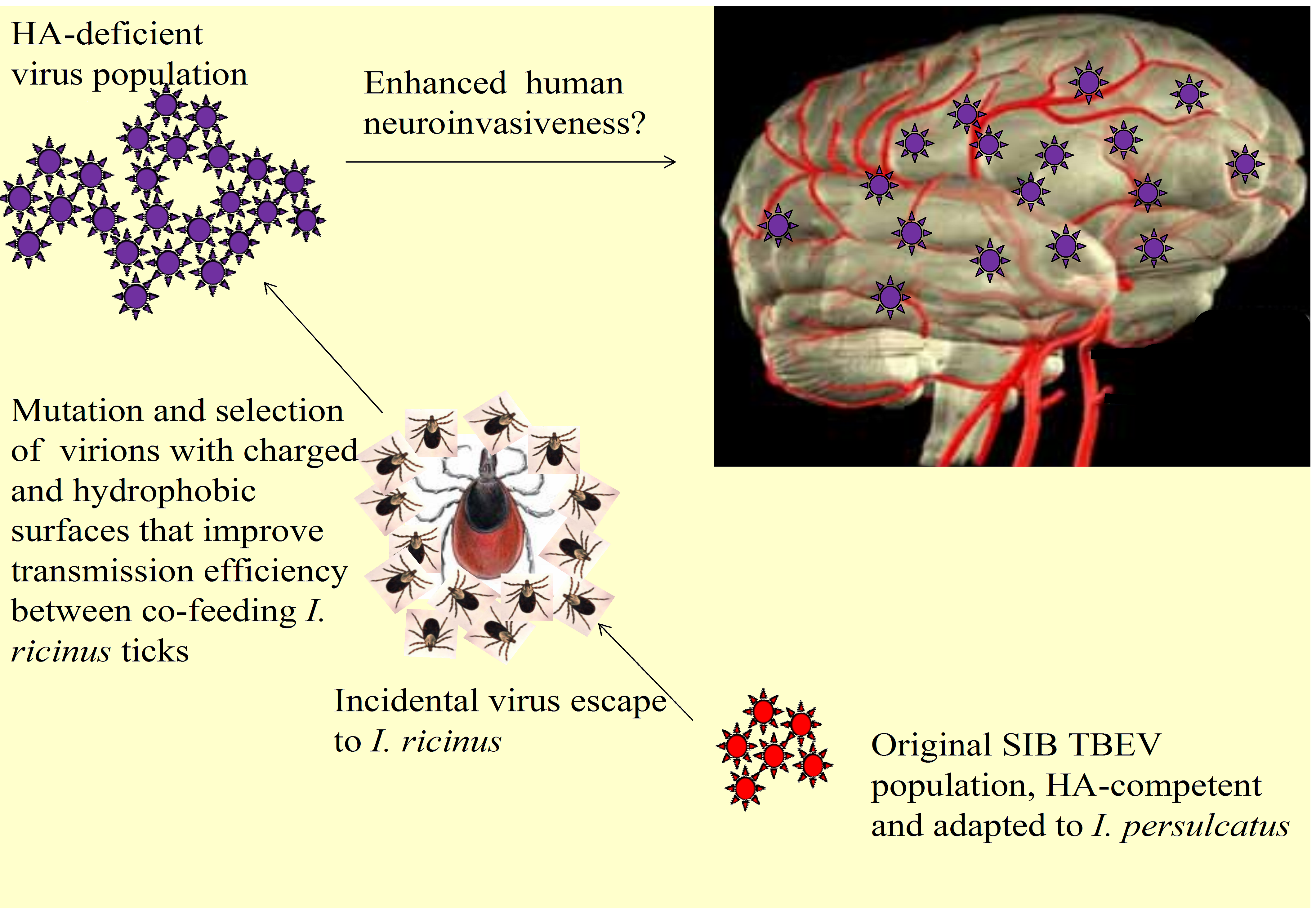

Supplement: Figure S1 — Cartoon illustrating the molecular mechanisms of emergence of new strains of TBEV with possible pathogenic characteristics for humans. (10.14 MB TIF) [file pone.0007295.s001.tif]
